# Leadership aspirations among residents in obstetrics and gynecology in the United States: a cross-sectional analysis

**DOI:** 10.1186/s12909-019-1757-x

**Published:** 2019-09-04

**Authors:** Brindha Bavan, Jordan Chavez, Briana Saravanabavanandhan, Jie Li, Shannon MacLaughlan David

**Affiliations:** 1 Department of Obstetrics and Gynecology, Stanford University School of Medicine, 300 Pasteur Drive, Stanford, CA 94305-5317 USA; 2 Stanford Health Care, Department of Social Work, 300 Pasteur Drive, Stanford, CA 94305-5617 USA; 3 Department of Graduate Medical Education, Stanford University School of Medicine, 300 Pasteur Drive - Room HC435, Stanford, CA 94305-5207 USA; 4 Department of Obstetrics and Gynecology, University of Illinois at Chicago, 1740 W Taylor St, Chicago, IL 60612 USA

**Keywords:** Leadership, Obstetrics, Gynecology, Residents, Gender, Mentorship, Graduate medical education

## Abstract

**Background:**

Most residents and faculty in obstetrics and gynecology (Ob/Gyn) are women. However, only a third of Ob/Gyn academic leadership positions are held by women in the United States.

**Methods:**

This is an IRB-approved cross-sectional study of leadership aspirations among Ob/Gyn residents in the U.S. as related to gender and mentorship using an electronic survey distributed nationwide in 2017. The primary outcome was resident interest in academic leadership. Mediator variables included demographics and training environment characteristics. Descriptive statistics and comparative analyses were performed using SPSS.

**Results:**

We received 202 completed surveys, for a representative cross-section of 4% of all Ob/Gyn residents in the U.S. The majority (86%) of respondents were women (*n* = 174), reflecting the same gender distribution of all Ob/Gyn residents in training. Sixty-seven percent of all respondents reported an interest in pursuing academic leadership (*n* = 133). Women reported leadership aspirations less often than men (64% vs 86%, *p* < 0.05) and reported lower mean Likert scores (3.73 vs 4.14, *p* < 0.05) regarding interest in leadership. A marginal difference between mean Likert scores was observed between women and men when controlled for other demographics (coefficient − 0.344, SE 0.186; *p* = 0.066). No difference in leadership aspirations was noted between women and men when controlled for mentorship, presence of female program director, and presence of three or more female leaders in a program.

**Conclusions:**

Gender disparity in goal-setting toward leadership is identified as early as residency training in Ob/Gyn. This imbalance in leadership aspirations can be addressed with targeted mentorship.

**Electronic supplementary material:**

The online version of this article (10.1186/s12909-019-1757-x) contains supplementary material, which is available to authorized users.

## Background

Women comprise about half of most graduating medical school classes in the United States, and now represent the majority of all students currently in medical school. However, women are still underrepresented in academic medicine, comprising only about a third of faculty [1, 2]. Furthermore, leadership positions such as chair, vice chair, and division director are disproportionately held by men. Even in the field of obstetrics and gynecology (Ob/Gyn), in which 85% of residents in the U.S. are female, only 21–35% of departmental academic leadership positions are held by women [1, 3].

Many possible explanations exist for this discrepancy. Gender biases and disparities have been measured in resident evaluations, faculty promotions, career development initiatives, and compensation [4–12]. However, there are no studies in the contemporary literature reporting on gender disparities among leadership aspirations of residents in Ob/Gyn in the U.S.

The purpose of this study is to evaluate the percentage of Ob/Gyn residents in the U.S. who identify academic leadership as a personal goal. We hypothesized that fewer women than men identify leadership as a goal during residency, and that women who do aspire to leadership are more likely to have had specific mentorship addressing leadership and have seen leadership modeled among women in their department.

## Methods

This cross-sectional study of academic leadership aspirations among Ob/Gyn residents in the U.S. was approved by the Stanford School of Medicine Institutional Review Board and was conducted using an electronic survey tool developed by our study team (see Additional file 1). The survey was distributed in 2017 to the U.S. Ob/Gyn Residency Program Coordinator Group ListServ, which was assembled and is maintained through the Association of Professors in Gynecology and Obstetrics. Residency program coordinators were asked to distribute the survey to their respective residents, who were incentivized to complete the survey by a chance to win one of five $100 Amazon gift cards. Survey data were collected over a period of 6 weeks and managed using the secure web application Research Electronic Data Capture (REDCap) hosted at Stanford University [13].

The primary outcome for this study was identification of leadership as a goal during residency. Respondents were asked to agree or disagree on a Likert scale (1 = strongly disagree, 2 = disagree, 3 = neutral, 4 = agree, and 5 = strongly agree) with the following statement: “It is a goal of mine to someday hold a leadership position (e.g. program director, department chair, society president).” The primary outcome was defined as an answer of agree or strongly agree for categorical descriptive analysis. Mean Likert scores were used for regression analyses.

Mediator variables included demographics (age, post-graduate year, relationship status, number of children) and current training environment (mentorship exposure and gender distribution within residency class, faculty, and academic leaders). It is important to note that responses to survey questions related to program environment are self-perceived by respondents and were not confirmed as factual data.

Descriptive and comparative (chi-square, T-test, F-test, and multivariate linear regression) analyses were performed using SPSS. A *p*-value of < 0.05 was designated as significant.

## Results

The survey was distributed to 241 residency program coordinators for dissemination to trainees. We received 202 completed surveys from residents across the U.S., representing 4% of the estimated 5187 Ob/Gyn residents in training at the time of the survey. The demographics of our cross-section is similar to what is known about the population of Ob/Gyn residents in general in the U.S. Specifically, the majority of respondents were women (86%, *n* = 174), the distribution of training years was even, and the majority of respondents were from university (71%, *n* = 143) or university-affiliated (16%, *n* = 32) programs [14].

All demographic characteristics are listed in Table 1.

**Table 1:** Demographic characteristics of survey respondents

Characteristic	n (%)
Gender	
Women	174 (86)
Men	28 (14)
Age (years)	
25–29	108 (53)
30–34	86 (43)
35–40	8 (4)
Post-graduate Year	
PGY-1	36 (18)
PGY-2	64 (32)
PGY-3	49 (24)
PGY-4	51 (25)
PGY-5+	2 (1)
Residency Setting	
University	143 (71)
University-affiliate	32 (16)
Community	27 (13)
Relationship Status	
Single	65 (32)
Married/Domestic Partnership	113 (56)
Other	23 (11.5)
Divorced/Separated	1 (0.5)
Children	
Yes	34 (17)
No	168 (83)

Sixty-seven percent of respondents (*n* = 133) identified leadership as a goal. Women were less likely than men to identify interest in leadership both when comparing total percentage (64% vs 86%, *p* < 0.05) and mean Likert scale responses (3.73 vs 4.14, *p* < 0.05). This gender disparity in responses persisted with marginal statistical significance (*p* = 0.066) after controlling for the effect of other demographic variables in a multivariate linear regression model (Table 2).

**Table 2:** Multivariate linear regression model of academic leadership interest by demographic characteristics

Characteristic	Unadjusted Mean	Coefficient ± SE	*P*-value
Gender			
Female	3.73	−0.344 ± 0.186	***p*** **= 0.66 (marginal)**
Male	4.14	–	
Age			
25–29	3.76	−0.407 ± 0.351	*p* > 0.1
30–34	3.80	0.248 ± 0.342	
35+	4.00	–	
Post-graduate Year			
PGY-1 and PGY-2	3.88	−0.237 ± 0.146	*p* > 0.05
PGY-3 and PGY-4	3.69	–	
Residency Setting			
University/university-affiliate	4.10	0.652 ± 0.188	***p*** **< 0.05**
Other	3.45	–	
Relationship Status			
Married/domestic partnership	3.66	−0.060 ± 0.209	*p* > 0.1
Single	4.03	0.204 ± 0.217	
Divorced/Other	3.71	–	
Children			
Yes	3.68	−0.072 ± 0.196	*p* > 0.1
No	3.81	–	
Constant	–	3.361 ± 0.398	*p* < 0.05

*SE* Standard Error

Statistically significant values in bold

About half of respondents reported receiving specific mentorship regarding leadership (Table 3). Sixty percent of participants had a mentor who was an academic leader him/herself. A majority of residents were from programs with mostly female trainees and mostly female or half female/half male faculty. Most residents who answered the survey had a female program director and clerkship director, but the minority of department chair positions were held by women, and only about a third of trainees reported their respective program having a total of three or more female academic leaders.

**Table 3:** Characteristics of current training environment

Characteristic	n (%)
Mentorship encouraging leadership	
Yes	99 (49)
No	103 (51)
Mentor who is a leader him/herself	
Yes	121 (60)
No	81 (40)
Residency Makeup	
Mostly to entirely women	199 (98.5)
50/50	3 (1.5)
Mostly to entirely men	0 (0)
Faculty Makeup	
Mostly to entirely women	90 (44.5)
50/50	106 (52.5)
Mostly to entirely men	6 (3)
Female Department Chair	
Yes	80 (40.5)
No	118 (59.5)
Female Program Director	
Yes	121 (60)
No	81 (40)
Female Clerkship Director	
Yes	143 (74)
No	50 (26)
Total number of female leaders	
0	30 (15)
1	44 (22)
2	66 (33)
3+	62 (30)

*F-test completed for this analysis

Four training environment factors correlated with a statistically significant increase in leadership interest (Table 4), including receiving direct mentorship about leadership (*p* < 0.01), having a mentor who is a leader him/herself (*p* < 0.01), having a female program director (*p* < 0.01), and having three or more academic leaders who are women in the department (*p* < 0.05).

**Table 4:** Effect of current training environment characteristics on academic leadership interest

Characteristic	Likert Mean	*P*-value*
Mentorship encouraging leadership		
Yes	4.11	***p*** **< 0.01**
No	3.48	
Mentor who is a leader him/herself		
Yes	3.92	***p*** **< 0.01**
No	3.59	
Residency makeup		
Mostly to entirely women	3.89	*p* > 0.1
50/50	4.33	
Mostly to entirely men	–	
Faculty makeup		
Mostly to entirely women	3.80	*p* > 0.1
50/50	3.78	
Mostly to entirely men	3.80	
Female Department Chair		
Yes	3.95	*p* > 0.1
No	3.67	
Female Program Director		
Yes	3.93	***p*** **< 0.01**
No	3.57	
Female Clerkship Director		
Yes	3.88	*p* > 0.1
No	3.56	
Total number of female leaders		
0	3.60	***p*** **< 0.05**
1	3.52	
2	3.76	
3+	4.10	

Statistically significant values in bold

In a multivariate linear regression model including all demographic and significant training environment characteristics, the effect of gender on interest in academic leadership weakened (*p* = 0.09). Training at a university or university-affiliate setting (*p* < 0.01) and receiving targeted mentorship about leadership (*p* < 0.05) remained statistically significant when controlling for all other factors (Table 5). Interestingly, only 46.4% of women (*n* = 79) reported being mentored regarding leadership, compared to 57.1% of men (*n* = 16) (*p* < 0.05).

**Table 5:** Multivariate linear regression model of academic leadership interest by all factors

Factor	Unadjusted Mean	Coefficient ± SE	*P*-value
Gender			
Female	3.73	−0.312 ± 0.181	*p* = 0.09
Male	4.14	–	
Age			
25–29	3.76	−0.311 ± 0.339	*p* > 0.1
30–34	3.80	0.183 ± 0.330	
35+	4.00	–	
Post-graduate Year			
PGY-1 and PGY-2	3.88	0.280 ± 0.139	*p* > 0.1
PGY-3 and PGY-4	3.69	–	
Residency Setting			
University/university-affiliate	4.10	0.417 ± 0.198	***p*** **< 0.05**
Other	3.45	–	
Relationship Status			
Married/domestic partnership	3.66	0.050 ± 0.199	*p* > 0.1
Single	4.03	0.338 ± 0.207	
Divorced/Other	3.71	–	
Children			
Yes	3.68	−0.090 ± 0.186	*p* > 0.1
No	3.81		
Mentorship encouraging leadership			
Yes	4.11	0.505 ± 0.128	***p*** **< 0.01**
No	3.48	–	
Mentor who is a leader him/herself			
Yes	3.92	0.195 ± 0.130	*p* > 0.1
No	3.59	–	
Female Program Director			
Yes	3.93	0.120 ± 0.194	*p* > 0.1
No	3.57	–	
Total number of female leaders			
0	3.60	–	*p* > 0.1
1	3.52	–	
2	3.76	–	
3+	4.10	0.071 ± 0.089	
Constant	–	3.361 ± 0.398	***p*** **< 0.05**

*SE* Standard Error

Statistically significant values in bold

Respondents were asked to agree or disagree with statements that may influence interest in pursuing leadership (Table 6). Over three-fourths of respondents cited personal career development (*n* = 151, 75%), the opportunity to mentor colleagues/trainees (*n* = 161, 80%), and the chance to positively impact the profession of Ob/Gyn at large (*n* = 173, 86%) as motivating factors for interest in academic leadership. Less than a third were incentivized by increased monetary compensation (*n* = 54, 27%). Additionally, one-third were concerned about interference with work/life balance (*n* = 66, 32%) and distraction from clinical practice (*n* = 67, 33%) as limiting factors. Of note, while targeted mentorship regarding leadership was a strongly associated factor in multivariate linear regression modeling, less than half of respondents (*n* = 99, 49%) agreed that receiving mentorship would motivate them to pursue leadership.

**Table 6:** Perceptions of Academic Leadership that Influence Interest

Factor	Agree n (%)	Neutral n (%)	Disagree n (%)
Motivating			
Focused mentorship about leadership	99 (4)	45 (22)	58 (29)
Positive examples of leaders within faculty	121 (60)	26 (13)	55 (27)
Personal career development	151 (75)	36 (18)	15 (7)
Prestige	60 (29.5)	61 (30.5)	81 (40)
Opportunity to mentor colleagues/trainees	161 (80)	14 (7)	7 (3)
Chance to impact profession of OB/GYN	173 (86)	23 (11.5)	5 (2.5)
Increase in total monetary compensation	54 (27)	94 (46)	54 (27)
Limiting			
Concern for interference with work/life balance	66 (32)	70 (34)	66 (32)
Distraction from clinical practice	67 (33)	76 (37)	65 (32)
Too much of a time commitment	59 (29)	78 (39)	65 (32)

## Discussion

We report that interest in leadership among Ob/Gyn residents correlates with trainees’ gender, training in a university or university-affiliate program, access to mentorship, and presence of female role models in leadership positions. A marginal gender disparity persists when controlled for other demographic characteristics of respondents, but this gender gap is closed when analysis is controlled for the effect of mentorship and presence of female role models. While this could be explained by an effect too small for detection in our study, it is also likely that mentorship and role modeling can negate the effects of gender bias in career development.

Our findings are congruent with other studies that have demonstrated gender disparities in academic leadership, promotion, and salaries. Significant gender differences in salary persist among faculty physicians in the U.S., even accounting for age, experience, markers of productivity, and academic rank [8, 15]. Additionally, women are less likely to attain senior level positions than male counterparts, even after adjusting for publication-related productivity [16]. This effect persists in the field of Ob/Gyn, in which women predominate the population of faculty, and yet the majority of leaders are male [1, 3, 17]. In a cross-sectional study of 950 academic medicine departments, Hofler et al. showed that Ob/Gyn departments had more women in leadership than other specialties in 2013. However, when considering the ratio of leaders to the number of women in the field, women in Ob/Gyn continue to be underrepresented at the leadership level compared to other specialties [1]. Our results suggest that a contributing factor to these disparities is that men identify leadership as a specific goal more often while in training, and therefore, initiate a targeted career development path sooner than women.

Our study also suggests that mentorship and female role modeling in leadership can address this gender gap. Studies in other fields have found that academic departments led by female department chairs were more likely to hire and promote female physicians and program directors through mentorship [18]. Mentorship has also been shown to influence career decision-making in Ob/Gyn. Cain et al. reported that 43% of female residents perceived that men were mentored and recruited more than women for faculty positions [19]. We note in our study that 46% of women reported they had received mentorship regarding leadership, compared to 57% of men, suggesting the presence of gender bias among mentors (*p* < 0.05).

We cannot assess presence of gender bias in faculty, staff, or other colleagues of our respondents with our survey tool. However, gender bias in resident evaluations and other assessments in career development and advancement is well documented [7, 11, 12, 20, 21]. A recent qualitative analysis of attending physician evaluations of residents in emergency medicine suggested that assertive characteristics were judged differently between men and women [11]. Similarly, among junior residents in Ob/Gyn, women received harsher feedback from labor and delivery nurses than men [7]. We suggest that this intrinsic bias in evaluators may influence leadership aspirations among women and may contribute to disparities observed or perceived in mentorship.

Addressing explicit and implicit gender biases with targeted interventions can reduce gender gaps in evaluation and promotion. For example, the University of California, Davis School of Medicine initiated training of search committees and promotion panels in unconscious bias and made significant progress in promoting women. The University of Massachusetts Medical School has increased the number of female full professors through mentoring and awards programs to support career development for women [22]. Increasing the number of professors and leaders who are women will improve trainees’ exposure to female role models, which was found to be a significant contributor to leadership aspirations in our study.

We are cautious in drawing conclusions from a cross-sectional study, as survey-based methodology cannot demonstrate causality and comes with intrinsic biases. Here we opted for a method of distribution of our survey that included all programs in the U.S., thereby, eliminating the selection bias associated with inviting “representative” programs for participation. This is a strength of our study. However, our method of distribution coupled with its anonymity does not allow us to measure a true survey response rate or describe non-responders. While our cohort of respondents is only 4% of the entire population of Ob/Gyn residents in the U.S., we believe it reflects a representative cross-section of the population we are studying, as demonstrated by the matching characteristics noted in demographics (e.g., gender distribution).

Given the introductory nature of this work, we intentionally kept our survey brief. We did not collect data on racial/ethnic background, a known contributor to perceptions and experiences in career development [19, 23, 24]. While we queried objective characteristics of training environments (e.g., university/university-affiliate versus community program), we did not ask about perceptions of the workplace environment, which is known to influence women in their career trajectories [10, 23–25]. We anticipate including these important mediator variables in a survey distributed directly to residents and fellows in training.

## Conclusions

To our knowledge, this is the first cross-sectional analysis of current Ob/Gyn residents in the U.S. and their aspirations toward leadership. By evaluating correlations here, we can consider causal relationships between existing systems in training programs and gender equality in academic medical leadership. We identified gender disparity in leadership goal setting as early as residency training. The effect of targeted mentorship, presence of female role models in leadership, and implementation of education around unconscious bias in residency curricula should be further explored in follow up research studies as avenues for overcoming barriers for women and men alike.

## Additional file

Additional file 1: Survey Questionnaire. (PDF 56 kb)

## Data Availability

The datasets used and/or analyzed during the current study are available from the corresponding author on reasonable request.

## Abbreviations

Ob/Gyn: Obstetrics and gynecology
REDCap: Research Electronic Data Capture
