# Supplementary material for: Leadership aspirations among residents in obstetrics and gynecology in the United States: a cross-sectional analysis
Source: BMC Med Educ. 2019 Sep 4;19:332. doi: 10.1186/s12909-019-1757-x (PMC6727386; doi:10.1186/s12909-019-1757-x)
Supplement: Supplementary file 1 — Survey Questionnaire. (PDF 56 kb) [file 12909_2019_1757_MOESM1_ESM.pdf]

Career Aspirations Among Ob/Gyn Residents

Dear OB/GYN Resident,

We invite you to participate in our survey evaluating career goals and aspirations among OB/GYN residents across the country.

We expect it will take 3-5 minutes of your time to complete the survey, and in return you can enter into a raffle to win one of five $100 Amazon gift cards. This survey is part of a research project approved by the Stanford University School of Medicine IRB. Your responses are completely anonymous and cannot be traced to you, your email address, or your residency program.

Thank you very much for your time!

ABOUT YOU...

Which of the following best describes your residency program?

- ◯ University
- ◯ University-affiliate
- ◯ Community hospital

Year of Residency

- ◯ Resident Year 1
- ◯ Resident Year 2
- ◯ Resident Year 3
- ◯ Resident Year 4
- ◯ Other (please specify)

Other

____

Age

____

Gender

- ◯ Female
- ◯ Male
- ◯ Transgender, female to male
- ◯ Transgender, male to female
- ◯ Gender non-conforming

Relationship Status

- ◯ Single
- ◯ Married/domestic partnership
- ◯ Other relationship
- ◯ Divorced/separated

Do you have children?

- ◯ Yes
- ◯ No

ABOUT YOUR GOALS...

What are your plans immediately after residency?

- ◯ Practice general OB/GYN
- ◯ Fellowship
- ◯ Other (please specify)

Other

____

In which environment do you see yourself ultimately practicing OB/GYN?

- ◯ University setting
- ◯ University-affiliate
- ◯ Community hospital
- ◯ Private practice
- ◯ Other (please specify)

Other ______________________________

Please rate how much you agree or disagree with the following statements as they relate to your personal goals in pursuing leadership roles (eg, program director, department chair, society president, etc):

	Strongly Disagree	Disagree	Neutral	Agree	Strongly Agree
It is a goal of mine to someday hold a leadership position.	◯	◯	◯	◯	◯
I have never considered professional leadership roles before.	◯	◯	◯	◯	◯
It is too early for me to be thinking about leadership roles.	◯	◯	◯	◯	◯

How likely are you to dedicate professional time after residency/fellowship graduation to each of the following?

	Not at all	Somewhat	Likely	Very likely
Clinical practice	◯	◯	◯	◯
Education for fellows/residents/medical students	◯	◯	◯	◯
Research	◯	◯	◯	◯
Administration	◯	◯	◯	◯
Other (please describe)	◯	◯	◯	◯

Other ______________________________

How likely are you to pursue each of the following leadership roles someday?

	Not at all	Somewhat	Likely	Very likely
Department Chair	◯	◯	◯	◯
Division Director	◯	◯	◯	◯
Fellowship/Residency Program Director	◯	◯	◯	◯
Medical Student Clerkship Director	◯	◯	◯	◯
Leadership role in professional society or organization (eg, ACOG)	◯	◯	◯	◯
Hospital Chief-Of-Staff	◯	◯	◯	◯
Hospital Administration	◯	◯	◯	◯
Other (please describe)	◯	◯	◯	◯

Other

____

How much mentoring have you received about pursuing each of the following leadership roles?

	None at all	Minimal	Adequate	Extensive
Department Chair	◯	◯	◯	◯
Division Director	◯	◯	◯	◯
Fellowship/Residency Program Director	◯	◯	◯	◯
Medical Student Clerkship Director	◯	◯	◯	◯
Leadership role in professional society or organization (eg, ACOG)	◯	◯	◯	◯
Hospital Chief-Of-Staff	◯	◯	◯	◯
Hospital Administration	◯	◯	◯	◯
Other (please describe)	◯	◯	◯	◯

Other

____

Please rate how much you agree or disagree with the following statements as they relate to your personal goals in pursuing leadership roles:

	Strongly Disagree	Disagree	Neutral	Agree	Strongly Agree
I would consider a leadership role in the future based on its prestige.	◯	◯	◯	◯	◯
I would consider a leadership position important for my personal career development.	◯	◯	◯	◯	◯
My mentors have encouraged me to consider leadership positions in my career.	◯	◯	◯	◯	◯
The mentor with whom I most closely identify holds a leadership position.	◯	◯	◯	◯	◯
Holding a leadership position would interfere with my work/life balance.	◯	◯	◯	◯	◯
Holding a leadership position would give me the opportunity to mentor colleagues and trainees.	◯	◯	◯	◯	◯
Holding a leadership position would give me the chance to impact the profession of OB/GYN.	◯	◯	◯	◯	◯
Holding a leadership position would detract from my clinical practice.	◯	◯	◯	◯	◯
Holding a leadership position is too much of a time commitment.	◯	◯	◯	◯	◯
Holding a leadership position would increase my total monetary compensation.	◯	◯	◯	◯	◯

ABOUT YOUR INSTITUTION...

Which of the following best describes the make-up of your residency class?

◯ Entirely women
◯ Mostly women
◯ 50% men and 50% women
◯ Mostly men
◯ Entirely men

Which of the following best describes the make-up of your residency overall?

- ◯ Entirely women
- ◯ Mostly women
- ◯ 50% men and 50% women
- ◯ Mostly men
- ◯ Entirely men

Which of the following best describes the make-up of your faculty?

- ◯ Entirely women
- ◯ Mostly women
- ◯ 50% men and 50% women
- ◯ Mostly men
- ◯ Entirely men

Does your institution offer any of the following training programs? (Check all that apply.)

- ☐ Maternal Fetal Medicine
- ☐ Reproductive Endocrinology and Infertility
- ☐ Gynecologic Oncology
- ☐ Urogynecology
- ☐ Family Planning
- ☐ Minimally Invasive Surgery
- ☐ Other (please describe)
- ☐ My residency program does not have any fellowship training programs

Other

____

Please indicate which of the following are included in your residency. (Check all that apply.)

- ☐ A structured mentorship program in which you have a designated individual mentor to address career development with you.
- ☐ Faculty members that hold leadership positions in professional societies and/or organizations (eg, ACOG, APGO).
- ☐ Independently funded faculty researchers (eg, principal investigators, NIH grant-holders, lab directors)
- ☐ None of the above

Please identify the gender of each of the following at your institution (Female, Male, or Not applicable/Unknown).

	Female	Male	Not applicable/Unknown
Department Chair	◯	◯	◯
Residency Program Director	◯	◯	◯
Medical Student Clerkship Director	◯	◯	◯
Hospital Chief-Of-Staff	◯	◯	◯
Hospital CEO	◯	◯	◯

Please estimate the percentage of Division Director positions (eg, service chief) in your department held by women.

- ◯ None
- ◯ < 10%
- ◯ 10-25%
- ◯ 25-50%
- ◯ 50-75%
- ◯ 75-100%
- ◯ unknown

Please estimate the percentage of Fellowship Director positions in your department held by women.

- ◯ None
- ◯ < 10%
- ◯ 10-25%
- ◯ 25-50%
- ◯ 50-75%
- ◯ 75-100%
- ◯ unknown

Please estimate the percentage of principal investigators/researchers in your department who are women.

- ◯ None
- ◯ < 10%
- ◯ 10-25%
- ◯ 25-50%
- ◯ 50-75%
- ◯ 75-100%
- ◯ unknown

END OF SURVEY - THANK YOU FOR YOUR PARTICIPATION!
